# Laser Guidance in C-Arm Cone-Beam CT-Guided Radiofrequency Ablation of Osteoid Osteoma Reduces Fluoroscopy Time

**DOI:** 10.1007/s00270-016-1533-9

**Published:** 2016-12-09

**Authors:** Maarten W. Kroes, Wendy M. H. Busser, Yvonne L. Hoogeveen, Frank de Lange, Leo J. Schultze Kool

**Affiliations:** Department of Radiology and Nuclear Medicine, Radboud University Medical Center, P. O. Box 9101, 6500 HB Nijmegen, The Netherlands

**Keywords:** Cone-beam computed tomography, Osteoid osteoma, Radiofrequency ablation, Image-guided therapy, Fluoroscopy, Laser guidance

## Abstract

**Purpose:**

To assess whether laser guidance can reduce fluoroscopy and procedure time of cone-beam computed tomography (CBCT)-guided radiofrequency (RF) ablations of osteoid osteoma compared to freehand CBCT guidance.

**Materials and Methods:**

32 RF ablations were retrospectively analyzed, 17 laser-guided and 15 procedures using the freehand technique. Subgroup selection of 18 ablations in the hip–pelvic region with a similar degree of difficulty was used for a direct comparison. Data are presented as median (ranges).

**Results:**

Comparison of all 32 ablations resulted in fluoroscopy times of 365 s (193–878 s) for freehand and 186 s (75–587 s) for laser-guided procedures (*p* = 0.004). Corresponding procedure times were 56 min (35–97 min) and 52 min (30–85 min) (*p* = 0.355). The subgroup showed comparable target sizes, needle path lengths, and number of scans between groups. Fluoroscopy times were lower for laser-guided procedures, 215 s (75–413 s), compared to 384 s (193–878 s) for freehand (*p* = 0.012). Procedure times were comparable between groups, 51 min (30–72 min) for laser guidance and 58 min (35–79 min) for freehand (*p* = 0.172).

**Conclusion:**

Adding laser guidance to CBCT-guided osteoid osteoma RF ablations significantly reduced fluoroscopy time without increasing procedure time.

**Level of Evidence:**

Level 4, case series.

## Introduction

Osteoid osteoma is a benign bone tumor that typically occurs in the extremities of children and young adults [1]. The tumor presents with intense pain, which has been reported to be a result of the nidus [2], associated hyperostosis [3], or the neural elements in the reactive fibrous tissue [4]. Excision or destruction of the nidus has been proven to be curative [5, 6]. Traditionally, treatment involved open excision of the osteoid osteoma; however, this has been replaced by minimally invasive methods of nidus destructions to shorten hospital stay and recovery time [7]. Various minimally invasive techniques have been described, such as percutaneous core drilling, percutaneous radiofrequency (RF) ablation, and laser photocoagulation [5, 6, 8]. The difficulty in these treatments is visualizing and targeting the small nidus (usually smaller than 15 mm diameter). Guidance techniques are therefore essential during these minimally invasive treatments. The use of cone-beam computed tomography (CBCT) guidance for positioning RF needles in the nidus of the osteoid osteoma has been described previously [9]. Advantages of CBCT guidance compared to conventional CT guidance are improved patient access [10] and higher accuracy in double-oblique needle placements irrespective of the angle of the needle path planning and the level of user experience [11].

CBCT guidance uses fluoroscopy imaging superimposed on the CBCT scan with needle path planning to visualize the actual needle position [12]. Two C-arm geometry positions are used to guide the needle with fluoroscopy to the nidus, namely the entry point view and the progress view [10]. The entry point view is used to find the skin entry point and to position the needle in the planned angle. The space between the patient and the detector of the C-arm, however, is limited and makes it challenging to drill a hole through the bone to the nidus along the planned path. The progress view, perpendicular to the entry point view, visualizes the needle progression from skin entry point to the target in relation to the planned path. To guide the drill in a controlled fashion, it is necessary to switch multiple times between entry point and progress views. This method will most likely increase the required fluoroscopy time together with the radiation exposure to the operator. A possible solution could be the use of a laser guidance device. The laser guidance system visualizes the planned needle angulation and skin entry point. Combined with the depth information provided by the C-arm in progress view, all information for accurate drill progression is provided simultaneously.

The current retrospective study was performed to assess whether laser guidance in CBCT-guided RF ablation of osteoid osteomas can reduce the required fluoroscopy compared to CBCT-guided procedures using the freehand technique. We further assessed whether laser guidance can aid in optimizing these procedures in terms of procedure time.

## Materials and Methods

### Radiofrequency Ablation

All RF ablations were performed using CBCT guidance (Allura Xper FD20, Philips Healthcare, Best, The Netherlands) in the interventional suite. Patient preparation included general anesthesia, grounding pads placement, and sterile covering. Each procedure started with acquiring a CBCT scan with the location of the osteoid osteoma centered in the image field as much as possible.

In the reconstructed 3D volume, the nidus was identified and a safe straight needle path was planned from skin entry point towards the nidus. The navigation software (XperGuide, Philips Healthcare, Best, The Netherlands) projected this planned needle path on the fluoroscopy images during the procedure to provide real-time feedback on needle position and progression in relation to the planned path and target point.

The entry point view, in which skin entry point and target were superimposed, was used to locate the skin entry point and place the drill (OnControl Bone Lesion Biopsy System, Teleflex Medical, Morrisville, USA) in the angulation of the planned path [13]. Rotating the C-arm to progress view, perpendicular to the needle path, allowed visualization of the drill-tip in relation to the nidus.

**Fig. 1:**
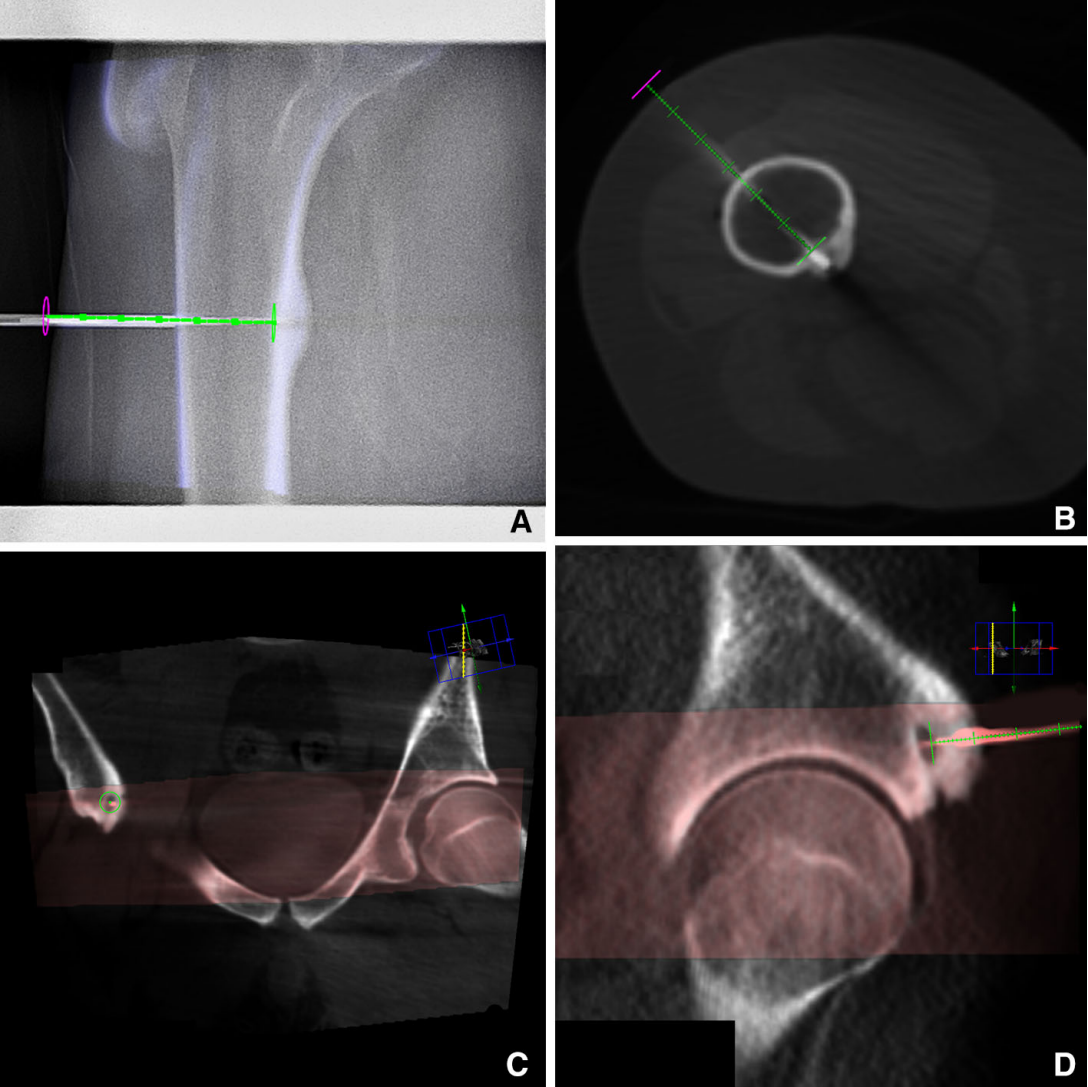
CBCT images of 2 patients with osteoid osteoma. **A** shows the overlay during fluoroscopy guidance in the progress view. **B** shows a CBCT scan of the same patient, with the RF electrode along the planned needle path (*green*). In **C** and **D**, the CBCT scan is fused with a previous CT scan, the CT scan is visualized in *gray* and the CBCT scan in *red*. In **C**, the entry *point view* is visualized and **D** shows the *progress view*

During drill progression, subsequent low-dose and collimated CBCT scans were acquired to confirm that the drill followed the planned needle path or to determine deviations from the path. These scans were acquired when desired by the performing interventional radiologist. In case of deviations caused by the amount of force put on the drill, the path of the drill was adjusted in a newly acquired CBCT scan. Figure 1 shows examples of image guidance and CBCT imaging.

After reaching the nidus, the drill was replaced by the RF electrode (StarBurst SDE 17 gauge, AngioDynamics, Latham, USA). To ensure that the tip was placed in the nidus, a final CBCT scan was acquired. The electrode tip temperature was increased to 85 °C and maintained for 4 min, ablating a sphere with <2 cm radius. After the ablation, the electrode was retrieved and the procedure ended.

### Laser Guidance

In procedures with laser guidance, placement and progression of the drill were assisted by a ceiling-mounted laser guidance unit (SimpliCT, Neorad AS, Oslo, Norway) acting as a laser pointing device.

**Fig. 2:**
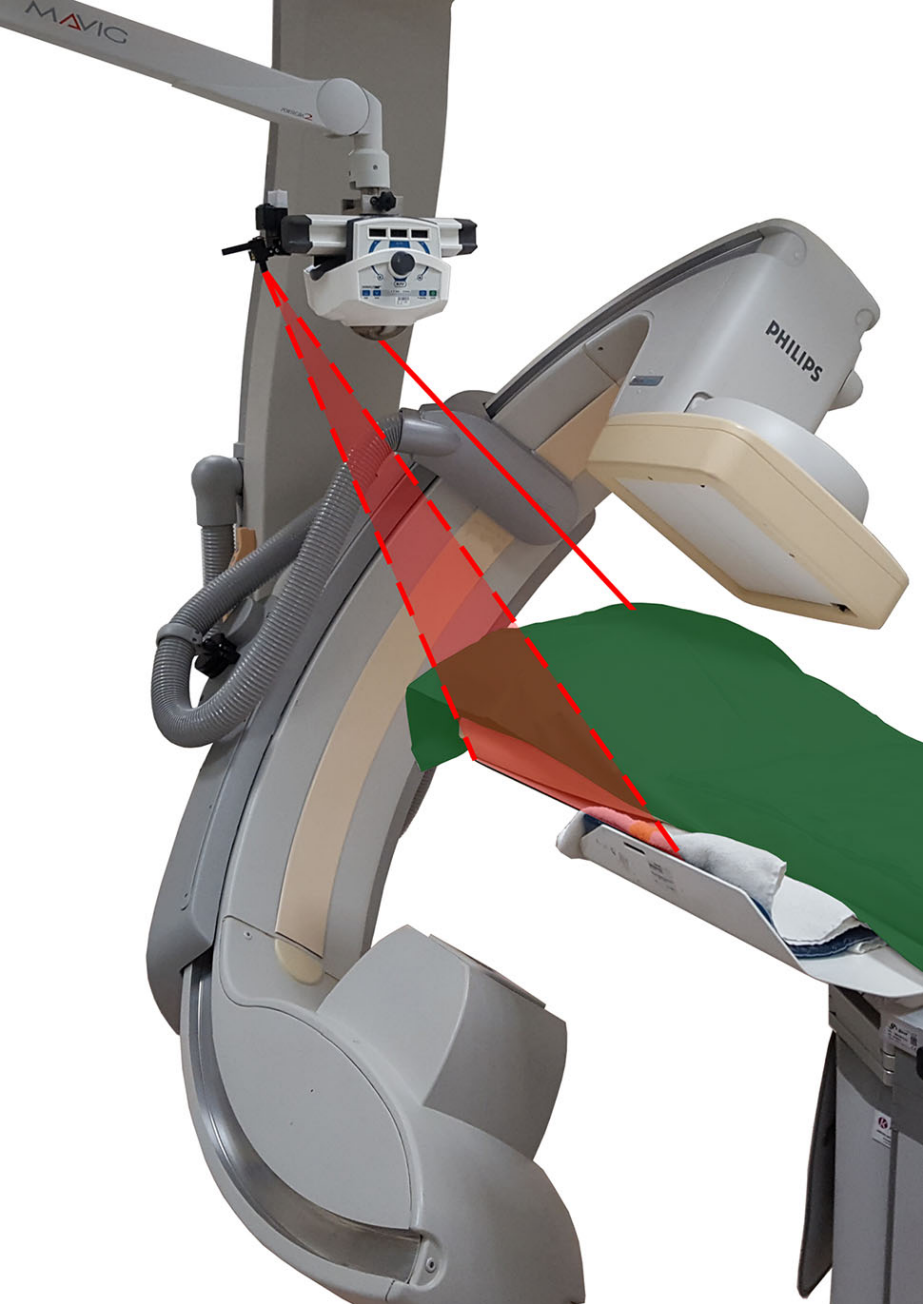
Schematic presentation of the laser guidance setup. The guiding laser from SimpliCT is aimed along a planned needle path (*straight line*), while the plane laser (*dotted lines*) is aligned to the operating table. The C-arm is positioned in *progress view*

**Fig. 3:**
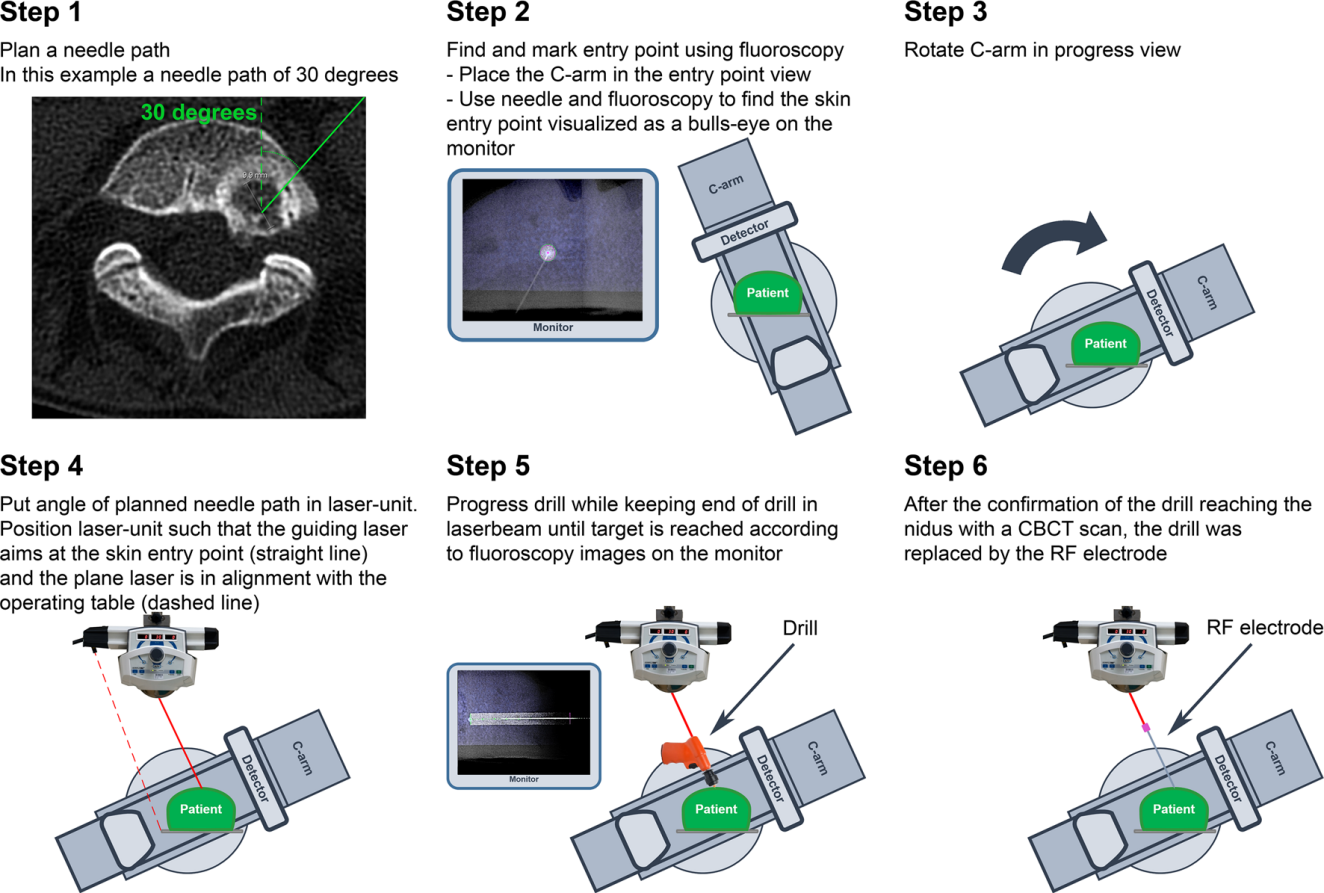
A detailed visualization of the steps for laser guidance during CBCT-guided RF ablation of osteoid osteoma

First, the skin entry point was located using fluoroscopy and marked with a marker. The C-arm was then positioned in the progress view, and the angles (in transversal plane and sagittal plane) of the needle path planning were fed into the laser unit. Hereafter, the pointing laser could be positioned on top of the marked skin entry point, while the plane laser was aligned with the operating table (Fig. 2). Using the laser pointer, the drill was placed in the angulation of the planned path. Throughout the drilling, the laser was used to keep the drill at the correct angulation, while fluoroscopy was used to check the depth of the drill. CBCT scans were acquired to confirm correct drill progression. The procedure including all laser guidance steps is visualized in Fig. 3.

### Protocol

This retrospective single-center study was exempted from approval by the institutional review board. The laser guidance device is commercially available and is a routinely used aid during our institution’s clinical–interventional CT procedures. Only when tumor location or patient positioning did not allow the use of laser guidance, CBCT guidance was used to position the drill on the target. The procedures were performed by three experienced interventional radiologists, all with 5 years or more experience in RF ablation for osteoid osteoma. Patients were included consecutively. Between June 2010 and January 2014, percutaneous RF ablation was performed in 32 patients with osteoid osteoma. In 17 cases, laser guidance was used as additional guidance tool in needle placement. In the other 15 cases, no additional needle guidance was used besides the CBCT guidance. Henceforth, the latter is referred to as freehand technique or freehand needle placement. Patient characteristics of both groups are provided in Table 1. This shows a large variety of locations for the osteoid osteoma. To accurately measure performance differences between both groups, a subgroup analysis was performed for 18 RF ablations with a similar degree of difficulty in the hip–pelvic region.

**Table 1:** Characteristics of osteoid osteoma patients

	Laser guidance	Freehand guidance
Number (male/female)	17 (14/3)	15 (10/5)
Age, median (range)	14 (4–34)	27 (9–55)
Location osteoid osteoma		
Hip–pelvic region	10	8
Tibia/fibula	4	2
Humerus	2	2
Tarsus	1	1
Ulna	0	1
Cervical vertebra	0	1

### Parameters and Analysis

Data of all parameters were retrospectively collected. Parameters provided by the imaging system were fluoroscopy time (in seconds), the number of acquired CBCT scans, and the dose area product (DAP) of fluoroscopy and CBCT scans (Gy cm^2^). The procedure time (in minutes) of the RF ablation was recorded, starting at the first CBCT scan and including laser setup time, and was stopped after the RF ablation was finished. Patient preparation and inducing anesthesia were not included. Technical success was defined as the RF needle tip positioned directly in the nidus and along the planned needle path. Length and height of all patients were converted into body-mass-index (BMI) data.

Statistical analyses were performed with IBM SPSS Statistics (v22.0; IBM Corporation, Armonk, USA). Differences between the two groups were analyzed using the Mann–Whitney *U* test. Two-sided *p* values ≤0.05 were considered statistically significant. Data are presented as medians with corresponding ranges.

## Results

**Fig. 4:**
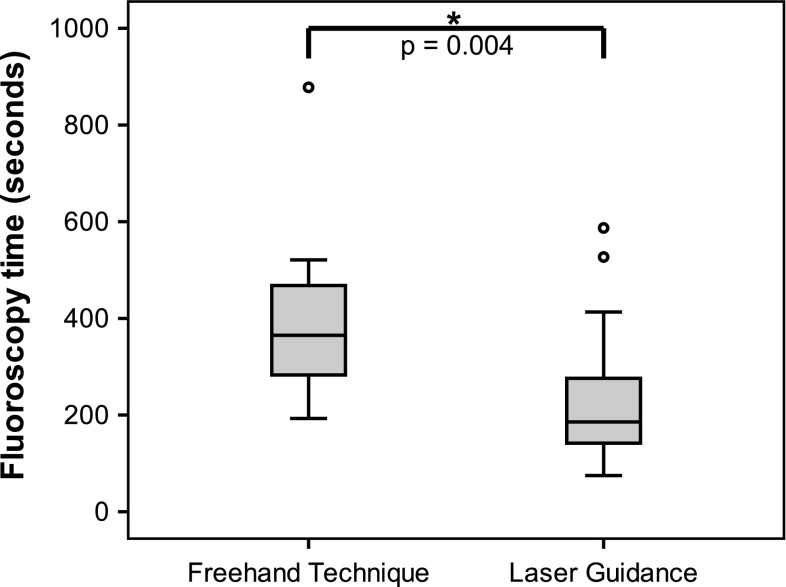
*Box plot* representing the fluoroscopy times in seconds required to guide the needle onto the target per guidance technique, with a significant reduction using laser guidance (*p* = 0.004)

Technical success was achieved in 100% of the RF ablations. For the total group of 32 RF ablations, adding laser guidance to the procedure resulted in a significant reduction of fluoroscopy time (*p* = 0.004). Fluoroscopy times were 186 s (75–587) vs. 365 s (193–878) for the laser procedures and the freehand procedures, respectively, indicating a reduction of 49% for laser-guided procedures (Fig. 4). Procedure times were not significantly different between the two groups, with 52 min (30–85) in the laser guidance group and 56 min (35–97) in the freehand group (*p* = 0.355).

The characteristics of the subgroup of 18 osteoid osteoma RF ablations in the hip–pelvic region are presented in Table 2. For both the laser guidance group and the freehand-guided group, we found similar target sizes (*p* = 0.965) and lengths of needle paths (*p* = 0.372). Comparing fluoroscopy times, laser guidance significantly reduced the number of fluoroscopy seconds (*p* = 0.012). A median of 215 s (75–413) was necessary to guide the drill, compared to 384 s (193–878) for the freehand group. For both the number of CBCT scans and procedure time, no statistically significant differences were found. The median number of CBCT scans for the laser guidance group was 4 (2–7) compared to 5 (3–7) for the freehand group. When laser guidance was used, the median procedure time was slightly lower with 51 min (30–72), while the median procedure time for the freehand group was 58 min (35–79).

**Table 2:** Characteristics of RF ablation of osteoid osteoma in hip–pelvic region

	Laser guidance	Freehand	*p* value
Osteoid osteoma locations	Femur: *n* = 9 Pelvic bone: *n* = 1	Femur: *n* = 5 Pelvic bone: *n* = 3	
Age (year)	11 (4–34)	26 (16–54)	0.011*
Diameter of irradiated anatomy (cm)	19.4 (16.2–28.3)	27.5 (12.4–31.9)	0.052
Target size (mm)	8 (6–10)	7.5 (6–10)	0.965
Length needle trajectory (mm)	54 (36–80)	65 (31–113)	0.372
Fluoroscopy time (sec)	215 (75–413)	384 (193–878)	0.012*
Percentage collimated fluoroscopy	44 (12–65)	38 (0–74)	0.657
No. of scans	4 (2–7)	5 (3–7)	0.308
Procedure time (min)	51 (30–72)	58 (35–79)	0.172

Data presented as median (range). The asterisk represents a statistically significant difference between the freehand technique and laser-guided procedures

For the freehand-positioned RF needles in the hip–pelvic region, the median fluoroscopy DAP value was 41.9 Gy cm^2^ (3.8–68.5). With a median of 2.8 Gy cm^2^ (1.6–27.1), fluoroscopy DAP for laser-guided RF ablations was significantly lower (*p* = 0.003). The CBCT DAP for the laser group was 9.3 Gy cm^2^ (1.7–77.2) vs. 35.5 Gy cm^2^ (3.7–91.2) for the freehand group (*p* = 0.068). The large differences in DAP compared to the differences in fluoroscopy time and number of CBCT scans are partly caused by the trend visible in the diameter size of the irradiated anatomy of the CBCT scan (*p* = 0.052). The trend of smaller patients for the laser group is caused by the statistically significant difference in age between the groups (*p* = 0.011), a median age of 11 years (4–34) compared to 26 years (16–54) for the freehand technique group. The difference in age is reflected in the BMI; for the laser group, the median BMI is 18 (14–24), while the fluoroscopy group has a median BMI of 25 (18–30) (*p* = 0.006).

## Discussion

The most essential finding of this study is that adding laser guidance to CBCT-guided RF ablation of osteoid osteoma significantly reduces fluoroscopy time, without extending the procedure time.

Fluoroscopy has been reported to be 35–45% of the total effective patient dose in CBCT-guided needle interventions [14, 15]. Reducing fluoroscopy time by employing laser guidance can serve to reduce the radiation exposure to the patient. Laser guidance was previously reported in CBCT-guided needle interventions in a laboratory setting using a phantom [16]. The results of the laboratory study showed a similar percentage of reduction in fluoroscopy time as the RF ablation in this clinical study. Here, the fluoroscopy time reduction itself is attributable to the visualization of the planned drill trajectory, leading to more efficient positioning of the drill, a reduced number of corrective manipulations of the drill, and less switching between entry point view and progress view as reflected by the significantly reduced time to guide the drill.

Overall, the number of necessary CBCT scans to complete the RF ablation procedures was higher compared to previously published CBCT-guided needle intervention studies that used 2–3 CBCT scans [14, 17, 18]. This difference could be explained by the difference in types of intervention. The referenced studies present data on biopsies in soft tissues, whereas the presented RF ablations comprise needle placements in bones. Due to the greater force required to drill through bone, extremities move or rotate and total patient position can shift. In these latter cases, the needle path planning based on the CBCT no longer matches with the new situation and a new CBCT is needed to adjust the planning. Patient fixation by a vacuum mattress could possibly reduce this effect.

Adding laser guidance to the procedure did not prolong total procedure time. Even though laser preparation and correct positioning take several minutes, this is compensated for by the time saved during the remainder of the procedure. Several other factors that could have influenced the procedure time can be identified, such as the ease of drilling towards the nidus, the number of CBCT scans acquired, how often the drill trajectory needs to be adjusted, and the length of the drill trajectory. These factors are all likely to be reflected in the wide range in recorded procedure times (30–79 min).

There are some limitations to the presented study. The retrospective nature of the study in combination with the relatively low number of 18 RF procedures available for subgroup analysis made it difficult to use the data for dose comparisons. To be able to show an effect on fluoroscopy DAP, a larger prospective study is required. For our single expert center, the number of patients which could be included is limited, as shown by the current study which spanned a period of 3 years; therefore, a multicenter prospective study is recommended. Even though fluoroscopy time is a direct measure for the number of needle manipulations and required time to position the needle tip in the target, fluoroscopy DAP also reflects differences related to collimation, choice of protocol, and size of irradiated volume. Therefore, the observed difference in DAP between groups could not be considered a direct effect of laser guidance. We did try to correct each DAP outcome for the differences in diameter size of irradiated anatomy, collimation per protocol used, number of frames per fluoroscopy second, and type of protocol to prove the effect of laser guidance in DAP. However, too many unknown variables have an effect on the outcome. We do know that the reduction in fluoroscopy times was a direct result of the use of laser guidance and reflected a relevant reduction in radiation exposure, irrespective of patient sizes or imaging protocols.

## Conclusion

Adding laser guidance to CBCT-guided osteoid osteoma RF ablation for drill alignment with the planned needle path significantly reduced fluoroscopy time up to 50%, without increasing procedure time.
